# Medical Mobile App Classification Using the National Institute for Health and Care Excellence Evidence Standards Framework for Digital Health Technologies: Interrater Reliability Study

**DOI:** 10.2196/17457

**Published:** 2020-06-05

**Authors:** Khine Nwe, Mark Erik Larsen, Natalie Nelissen, David Chi-Wai Wong

**Affiliations:** 1 Leeds Institute of Health Sciences University of Leeds Leeds United Kingdom; 2 Black Dog Institute University of New South Wales Sydney Australia; 3 Leeds Institute of Data Analytics University of Leeds Leeds United Kingdom; 4 Centre for Health Informatics University of Manchester Manchester United Kingdom; 5 Department of Computer Science University of Manchester Manchester United Kingdom

**Keywords:** mHealth, telehealth, evaluation, evidence, interrater, NHS Apps Library, NICE

## Abstract

**Background:**

Clinical governance of medical mobile apps is challenging, and there is currently no standard method for assessing the quality of such apps. In 2018, the National Institute for Health and Care Excellence (NICE) developed a framework for assessing the required level of evidence for digital health technologies (DHTs), as determined by their clinical function. The framework can potentially be used to assess mobile apps, which are a subset of DHTs. To be used reliably in this context, the framework must allow unambiguous classification of an app’s clinical function.

**Objective:**

The objective of this study was to determine whether mobile health apps could be reliably classified using the NICE evidence standards framework for DHTs.

**Methods:**

We manually extracted app titles, screenshots, and content descriptions for all apps listed on the National Health Service (NHS) Apps Library website on July 12, 2019; none of the apps were downloaded. Using this information, 2 mobile health (mHealth) researchers independently classified each app to one of the 4 functional tiers (ie, 1, 2, 3a, and 3b) described in the NICE digital technologies evaluation framework. Coders also answered contextual questions from the framework to identify whether apps were deemed to be higher risk. Agreement between coders was assessed using Cohen κ statistic.

**Results:**

In total, we assessed 76 apps from the NHS Apps Library. There was classification agreement for 42 apps. Of these, 0 apps were unanimously classified into Tier 1; 24, into Tier 2; 15, into Tier 3a; and 3, into Tier 3b. There was disagreement between coders in 34/76 cases (45%); interrater agreement was poor (Cohen κ=0.32, 95% CI 0.16-0.47). Further investigation of disagreements highlighted 5 main explanatory themes: apps that did not correspond to any tier, apps that corresponded to multiple tiers, ambiguous tier descriptions, ambiguous app descriptions, and coder error.

**Conclusions:**

The current iteration of the NICE evidence standards framework for DHTs did not allow mHealth researchers to consistently and unambiguously classify digital health mobile apps listed on the NHS app library according to their functional tier.

## Introduction

Mobile apps for use in health care have been proposed in a variety of settings, including telehealth for disease management and monitoring, diagnosis and triage, and medication prescription and reminders [1-3]. To date, the evidence for their effectiveness is varied [4-6] and, in general, published evidence is extremely sparse [7].

Thorough governance of medical apps is challenging [8]. There is growing recognition that the required level of evidence for medical apps ought to differ depending on app function. For instance, Wyatt [9] suggested that apps be categorized as low, medium, or high risk for purposes of evaluation. The UK National Institute for Health and Care Excellence (NICE) further addressed this issue through their recent evidence standards framework for evaluating digital health technologies (DHTs) [10]. In this framework, digital technologies are categorized into one of 3 tier levels (with Tier 3 split into Tier 3a and 3b) according to their intended function. An additional set of questions assesses higher-risk technologies, complementing the tier levels.

The framework also provides minimum and best-practice recommendations on the associated standard of evidence required for each tier. For instance, Tier 3a technologies should be supported, at a minimum, by relevant *high-quality observational* or *quasi-experimental studies.* Best practice would include a *high-quality intervention study*. The recommended evidence standards are cumulative, such that Tier 3 digital technologies should also meet the criteria for Tier 2 and Tier 1 technologies. For Tier 2, this includes, for instance, having evidence to show that any health information provided is valid, accurate, up-to-date, regularly audited, and sufficiently comprehensive. At the time of writing, the framework is in its second iteration.

Although other frameworks exist and have been used for classifying mobile apps [11], we solely examine the NICE framework here. This framework is of particular importance as its development was commissioned by the National Health Service (NHS) England and is therefore likely to become an influential standard. In addition, its recency means that it has thus far received little external validation.

This study aims to evaluate the appropriateness and potential limitations of the functional classification guidance within the NICE framework as applied to trusted and safe mobile health (mHealth) apps. We will do this by assessing interrater agreement of functional tier classification for all apps curated on the NHS Apps Library. By examining cases in which reviewers disagreed, we will highlight ambiguities in the current classification guidance and discuss potential improvements.

## Methods

### Search Strategy and Data Extraction

All apps available on the NHS Apps Library [12] on July 12, 2019, were included in the analysis. For each app, the app title, screenshots, and description were extracted manually from the NHS Apps Library website; none of the apps were downloaded. No apps were excluded.

### Classification

Two coders independently classified all apps according to functional classification. The coders were a clinician with formal postgraduate training in health informatics (KN) and an academic with research expertise in mHealth (MEL).

For each app, we recorded the main features as described on the NHS Apps Library, including any available screenshots. The coders assigned each app to a functional tier and noted whether the app should be considered for risk adjustment based on clinical context. Abridged information about each tier and criteria for determining risk-adjusted apps are shown in Tables 1 and 2. To guide our classification decisions, we used the (unabridged) evidence standards framework alongside the associated user guide [13]. If an app could be assigned into multiple tiers, it was assigned to the highest relevant tier, as per the framework guidance. If apps met the criteria for both Tier 3a and Tier 3b, they were assigned to 3b.

**Table 1:** Abridged definitions of digital health technology tiers from the National Institute for Health and Care Excellence (NICE) evidence standards evaluation framework [10].

Tier and functional classification		Description
**1**		
	System service	Improves system efficiency. Unlikely to have direct and measurable individual patient outcomes.
**2**		
	Information	Provides information and resources to patients or the public.
	Simple monitoring	Allows users to record health parameters to create health diaries.
	Communicate	Allows two-way communication between users and professionals, carers, third-party organizations, or peers.
**3a**		
	Preventative behavior change	Designed to change user behavior related to health issues with, for example, smoking, eating, alcohol, sexual health, sleeping, and exercise.
	Self-manage	Aims to help people with a diagnosed condition to manage their health.
**3b**		
	Treat	Provides treatment for a diagnosed condition (such as cognitive behavioral therapy for anxiety), or guides treatment decisions.
	Active monitoring	Automatically records information and transmits the data to a professional, carer, or third-party organization, without any input from the user, to inform clinical management decisions.
	Calculate	Tools that perform clinical calculations that are likely to affect clinical care decisions.
	Diagnose	Uses data to diagnose a condition in a patient, or to guide a diagnostic decision made by a health care professional.

**Table 2:** Abridged contextual questions to help identify higher-risk digital health technologies (DHTs), abridged from [10].

Question	Risk adjustment
1. Are the intended users of the DHT considered to be in a potentially vulnerable group such as children or at-risk adults?	The National Health Service (NHS) England defines an at-risk adult as an adult “who may be in need of community care services by reason of mental or other disability, age or illness; and who is or may be unable to take care of him or herself, or unable to protect him or herself against significant harm or exploitation.” If the DHT is intended to be used by people considered to be in a potentially vulnerable group, then a higher level of evidence may be needed, or relevant expert opinion on whether the needs of the users are being appropriately addressed.
2. How serious could the consequences be to the user if the DHT failed to perform as described?	A higher level of potential harm may indicate that the best-practice evidence standards should be used.
3. Is the DHT intended to be used with regular support from a suitably qualified and experienced health or social care professional?	DHTs that are intended to be used with support (ie, with regular support or guidance from a suitably qualified and experienced health or social care professional) could be considered to have lower risk than DHTs that are intended to be used by the patients on their own. This contextual question may require careful interpretation depending on the individual DHT as the involvement of a clinician may in itself indicate that the DHT presents a specific risk.
4. Does the DHT include machine learning algorithms or artificial intelligence?	Refer to the code of conduct for data-driven health and care technology for additional considerations when assessing DHTs that use artificial intelligence or machine learning.
5. Is the financial or organizational risk of the DHT expected to be very high?	DHTs with very high financial risk should be assessed using the best-practice standards to provide surety that the DHT represents good value. High organizational risks may include situations in which implementing the DHT would need complex changes in working practice or care pathways.

### Classification

We used descriptive statistics to summarize the membership of each tier and the differences in classification between reviewers. We reported Cohen κ as an overall measurement of interrater agreement [14]. In supplementary analysis, we reported interrater agreement for a subset of apps that had previously been classified by another independent team and published in the framework user guide. All analyses were conducted using MATLAB (version 18.1; MathWorks) [15].

We examined any apps with discrepancies in functional tier classification in greater detail. Using the publicly available descriptions of each app, we identified common themes that may have led to differences in classification.

## Results

### Interrater Agreement Results

In total, we assessed 76 apps from the NHS Apps Library. Overall, 0 apps were unanimously classified into Tier 1; 24/76 (32%), into Tier 2; 15/76 (20%), into Tier 3a; and 3/76 (4%), into Tier 3b. Full classification details for each app are presented in Multimedia Appendix 1.

Table 3 shows the interrater agreement for each tier of app. There was disagreement in 34/76 cases (45%) and Cohen κ was 0.32 (95% CI 0.16-0.47), which is commonly considered to correspond to poor agreement [12]. Of the 34 apps for which functional classification differed, 13 were due to discrepancies between apps classified in Tier 3a or 3b. The next largest group of discrepancies was with apps classified between Tiers 2 and 3a (n=11). Analysis of interrater agreement for a subset of apps previously reviewed by a third independent group is presented in Multimedia Appendix 2 (coder 1: κ=0.48, 95% CI 0.31-0.66; coder 2: κ=0.62, 95% CI 0.44-0.80). The apps for which the independent coders disagreed are also listed in Multimedia Appendices 1 and 2.

Coders also assessed on whether an app should be considered for *risk adjustment* or not based on a set on contextual questions (Table 2). Of these, Questions 2, 4, and 5 were not possible to answer without wider knowledge of the source code (for Question 4) or how the app interacted with the wider health care system. A total of 9 apps were unanimously considered to warrant risk adjustment, and 63 for no adjustment. There were discrepancies for 4 apps, and overall agreement may be considered good (κ=0.79; 95% CI 0.59-0.99).

**Table 3:** Number of apps coded into each functional tier classification, for both coders.

Classification: coder 1	Classification: coder 2								
	1		2		3a		3b		
1		0		6		0		0	
2		1		24		5		3	
3a		0		6		15		1	
3b		0		0		12		3	

### Analysis of Coder Discrepancies

On further collaborative review, differences in tier classification could be attributed to 5 causes: (1) app function not listed within framework, (2) app function corresponded to multiple tiers, (3) ambiguous tier description, (4) ambiguous app description, and (5) human error.

#### App Function Not Listed Within Framework

This occurred when the intended function of the app, as described on the NHS Apps Library, did not correspond to any of the functions listed within the NICE framework. One example of this was *MyChoicePad*, an app that is designed to assist nonverbal communication via symbols and signs from the Makaton language. Although one coder categorized the app and function as Tier 2: Communicate, the app does not facilitate two-way communication, so it does not strictly meet the associated criteria. Similarly, the other reviewers categorized the app into Tier 1: System service, but it is not entirely clear whether the app is designed to improve system efficiency, or even what the system is in this case.

#### App Function Corresponded to Multiple Tiers

This occurred when a single function of an app corresponded to more than 1 tier within the NICE framework. In particular, we noted ambiguity around mental health apps. For example, mindfulness or principles from cognitive behavioral therapy may be classified as a self-management strategy to reduce feelings of anxiety (3a), or as a treatment for anxiety disorders (3b). In this situation, the tier classification depended on the clinical use case, rather than the app function, which was the same in both cases. We also noted ambiguity between lifestyle/well-being versus diagnosed conditions. For example, an app that had a function to provide advice on stress or anxiety reduction might be classified into Tier 2 if it provides generic information, but into 3a if *stress* were part of a diagnosed condition.

#### Ambiguous Tier Description

Some terms used to describe the tiers within the NICE framework were not clearly defined. One specific instance of ambiguity between Tiers 3a and 3b occurred for the *engage warfarin self-care* app. The app allows warfarin test results to be *actively reviewed* by a clinician, but it is unclear whether this constitutes Tier 3b’s *active monitoring* as results must be manually transmitted by the user, or *simple monitoring* which allows users to record their health parameters.

#### Ambiguous App Description

In some instances, the information provided on the NHS Apps Library was insufficient to definitively categorize an app. For example, screenshots on the NHS Apps Library for the *NHS App* show that users can *check symptoms,* but this functionality was not mentioned elsewhere. The main description for the app stated that users can “find reliable NHS information on hundreds of conditions and treatments, and get immediate advice.” If the app merely catalogs searchable information on symptoms, it should be classed as Tier 2; however, if it provides a symptom checker algorithm that requires user input, and outputs relevant immediate advice, it is Tier 3b.

#### Human Error

This occurred when the reviewer failed to identify a relevant piece of information that would have influenced their tier categorization decision. This most commonly occurred when an app had multiple functions belonging to multiple functional tiers. Typically, the main stated function of the app belonged to the lower tier. For instance, the *Healthera* app is primarily designed to manage prescription management, a Tier 1 function. However, it also allows users to contact their pharmacist for clinical advice via the app, a Tier 2 function. Human errors accounted for 6 apps. In conjunction with the primary result, we can estimate an upper bound on the level of disagreement as 28/76 (37%; κ=0.44, 95% CI 0.30-0.60), when human error is removed.

## Discussion

### Principal Results

Our results show that, from their publicly available descriptions, only 42 of the 76 apps collated in the NHS Apps Library could be classified into a functional tier consistently by informed individuals. Interrater agreement between reviewers was poor (κ=0.32).

Of the 34 apps where there was disagreement, there was a subset (28/34) for which the tier could not be agreed, even after consultation (ie, excluding human error). In these cases, disagreement was attributed to four broad categories: *App function not listed within framework*, *App function corresponded to multiple tiers*, *Ambiguous tier description*, and *Ambiguous app description*.

There was good agreement in assessment of *higher risk* using the NICE framework’s contextual questions (κ=0.79). Despite this, we noted ambiguity in the risk adjustment questions. One example of ambiguity occurs for the assessment question: “Does the DHT include machine learning algorithms or artificial intelligence?” If yes, framework users are advised to refer to the Code of Conduct for data-driven health and care technology [16].

Whilst the framework defines both artificial intelligence and machine learning, the provided definitions are ambiguous. For instance, it states that “Machine learning is an application of artificial intelligence that provides systems the ability to automatically learn and improve from experience without being explicitly programmed.” This definition would include the vast majority of quantitative methods. For instance, linear regression is fully defined by the slope and offset parameters, as learned directly from a data set.

The Code of Conduct further provides an external link to the definition from the AHSN Network AI Initiative [17]. No definition is provided here, instead readers are informed that “there is no single, universally agreed definition of AI.” The absence of any clear definition means that this risk adjustment question cannot be answered objectively.

Poor interrater agreement of tier classification may be attributed to two potential causes. First, the publicly available information on the NHS App Library may be insufficient to determine the functional tier. If true, this would motivate tighter regulation of how apps are described on the Library to ensure that the intended medical condition and patient or user group is clear. Second, the DHT framework is not specific enough to classify some types of apps. Based on our thematic analysis, in which we showed examples of misclassified apps due to ambiguity in the framework, we contend that this second reason contributes significantly to the overall level of disagreement.

A framework with inadequate specificity has implications for both developers and regulators. For developers wishing to bring products to market as soon as possible, opportunity for misclassification due to ambiguous tier criteria may result in more classifications to lower tiers, where the minimum standard of evidence is not as stringent. In particular, Tier 2 technologies require only information that would be commonly audited in standard software development, whereas Tier 3a and Tier 3b technologies specifically require formal studies that would likely require additional time and financial resource. This may lead to situations in which apps are regulated to a level of lower scrutiny than they ought to be, given their function.

In addition to the main result, we observed that some apps were categorized consistently by reviewers, but the minimum suggested level of evidence did not seem to align with the level of potential clinical risk. One example of this was *Cypher*, which was classified in Tier 2 as it facilitates communication. Whereas other Tier 2 apps allowed communication with health professionals, this app facilitates anonymous communication with other users to allow “anyone who want to share their thoughts”. (We further note that as of October 17, 2019, Cypher App is not available for download on the either the Google Play store or Apple App Store and that the developer website is nonfunctional. Persistence of apps is a known problem in digital health [18].) The evidence framework directly addresses this use case by requiring peer-support apps to show evidence of appropriate safeguarding. However, internet communities, forums, and chat rooms [19,20] with similar peer-support functions have been shown to have potential for negative patient impact. Given that the potential harm aligns more closely with apps in Tier 3a or 3b, a higher standard of evidence may be appropriate for peer-support apps, despite the similarity in technical function to other communication apps.

Within mHealth more widely, we can select examples in which the dissonance between functional tier and required evidence is even greater. For instance, consider an app that calculates BMI by requiring the user to type height and weight. This may be categorized into Tier 3b, as a tool that performs a clinical calculation that can affect clinical care decisions. The associated minimum standard of evidence in the NICE framework requires a high-quality intervention study—a level of scrutiny that ought not to be required for simple and well-established calculations. This does not preclude the need for careful technical evaluation; indeed, Huckvale et al [21] have demonstrated how even simple clinical calculations in diabetes apps are often calculated or displayed incorrectly.

More broadly, this indicates that clinical risk and technical risk are not necessarily the same. One possible option to enable better classification and more specific evaluation guidance may be to categorize apps by technical complexity as well as clinical function. The idea of separating clinical and technical evaluation has been raised previously. Lewis and Wyatt [22] suggest evaluation could be based on the probability and severity of clinical harm, the complexity of the app, and additional contextual factors [22]. Others have commented that evidence of clinical effectiveness for software should include indication of safety, and that this must include formal technical evaluation [23].

### Limitations

The NICE framework is designed for DHTs that are commissioned by the health system. In this case, we examined apps on the NHS Apps Library. According to the library’s HTML description metadata, these are “digital tools that have been assessed by the NHS as clinically safe and secure to use” [12]. However, in most instances, the apps had not been specifically commissioned. Although the relevance of the framework for these apps may vary, multiple apps in the library (eg, GDm-Health, Health Help Now) have already been used as part of routine clinical care.

The primary limitation of work is that our classifications relied on the information presented by the NHS App Library; additionally, none of the apps were downloaded. As all products published in the Library met a set of internal standards, we believed, a priori, that written descriptions and screenshots should be sufficient to enable clear identification of all key functions (as this is key information for informing consumer app selection). Our results showed that this was not the case. Downloading each app would provide more comprehensive understanding of the key functions and may increase interrater agreement on app tier. We therefore recommend that future studies download and engage directly with the contents of mobile apps.

Our study compares only two sets of raters, so the results might be unduly influenced by the poor performance of a single individual. We partly addressed this by comparing interrater reliability with a third set of reviewers (see Multimedia Appendices 1 and 2), for which similar results were obtained. These were not included in the main manuscript as reviews were conducted under different conditions; we do not know if apps were downloaded, nor the time at which their review took place.

Finally, the NICE framework establishes a functional tier and provides guidance on required levels of evidence at each tier. In this work, we only examined the consistency of tier classification, and did not address whether apps within a tier met the evidence standards.

### Conclusions

The NICE evidence standards framework for evaluating digital technologies is a significant and timely step toward establishing appropriate levels of evidence for DHTs. Despite this, we have demonstrated that the current iteration of the framework did not allow mHealth researchers to consistently and unambiguously group a set of digital health mobile apps according to their functional tier. In users with limited experience of mHealth evaluation (eg, app developers), we postulate that this ambiguity may lead to higher levels of misclassification. One potential improvement would be to classify DHTs by their technical complexity in addition to clinical function.

## Appendix

Multimedia Appendix 1 Raw data used for analysis. Independent data were extracted from NICE case studies.

Multimedia Appendix 2 Inter-rater reliability crosstables for coders 1 and 2 vs the case study examples provided with the evidence standards framework.

## Abbreviations

DHT: digital health technologies
NHS: National Health Service
NICE: National Institute for Clinical Excellence
