# Supplementary material for: Medical Mobile App Classification Using the National Institute for Health and Care Excellence Evidence Standards Framework for Digital Health Technologies: Interrater Reliability Study
Source: J Med Internet Res. 2020 Jun 5;22(6):e17457. doi: 10.2196/17457 (PMC7305556; doi:10.2196/17457)
Supplement: Multimedia Appendix 1 [file jmir_v22i6e17457_app1.docx]

## Multimedia Appendix 1

Raw data used for analysis. *Independent* data was extracted from <https://www.nice.org.uk/Media/Default/About/what-we-do/our-programmes/evidence-standards-framework/functional-classification-case-studies.pdf> (accessed 01/12/2019)

| **App Name** | Coder 1 | Coder 2 | Independent |
| --- | --- | --- | --- |
| Baby and Child First Aid | 2 | 2 | 2 |
| Baby Buddy | 2 | 2 | 2 |
| Be Mindful | 3a | 3b | 3b |
| Beat Panic | 3a | 3b | 3a |
| BECCA Breast Cancer Care App | 2 | 2 | 2 |
| Big White Wall | 3b | 2 | 3b |
| BlueIce | 3a | 3b | 3a |
| Brush DJ | 2 | 2 | 2 |
| Calm Harm | 3a | 3b |  |
| Catch It | 3a | 3b |  |
| Changing Health | 3a | 3a | 3a |
| ChatHealth | 2 | 2 | 2 |
| Chill Panda | 3a | 3a | 3a |
| Co-op Health | 2 | 1 |  |
| Cove | 2 | 2 | 3a |
| Cypher | 2 | 2 |  |
| distract | 2 | 2 |  |
| Echo | 2 | 1 | 2 |
| Engage Consult | 2 | 2 | 2 |
| engage warfarin self-care | 3a | 3b | 3a |
| Evergreen Life | 2 | 2 | 2 |
| Famiy Assist | 2 | 2 |  |
| Feeling Good: positive mindset | 3a | 3b | 3a |
| First Aid by British Red Cross | 2 | 2 | 2 |
| GDm-Health | 3b | 3b | 3b |
| Health Help Now | 3b | 2 | 2 |
| Healthera | 2 | 1 | 2 |
| HealthUnlocked | 2 | 2 | 2 |
| Hoop | 2 | 1 |  |
| Ieso | 3a | 2 | 2 |
| Integrated Family Delivered Neonatal Care (IFDC) | 2 | 2 | 2 |
| iPrescribe Exercise | 3a | 3b | 3a |
| Kicks Count | 2 | 2 | 2 |
| Liva UK | 3a | 3a | 3a |
| Low Carb Program | 3a | 3a |  |
| mapmydiabetes | 3a | 3a | 3a |
| MeeTwo | 2 | 2 |  |
| Mumoactive | 3a | 3a | 3a |
| My Diabetes My Way | 2 | 2 | 3a |
| My Health Fabric | 3a | 3a | 3a |
| My Health Guide | 3a | 2 | 2 |
| My House of Memories | 3a | 2 |  |
| my mhealth: myAsthma | 3a | 3a |  |
| my mhealth: myCOPD | 3a | 3a | 3a |
| my mhealth: myDiabetes | 3a | 3a |  |
| my mhealth: myHeart | 3a | 3a |  |
| My Possible Self: The Mental Health App | 3a | 3b | 3a |
| MyChoicePad | 2 | 1 | 2 |
| MyCognition Home | 3a | 3b | 3a |
| myGP | 2 | 2 |  |
| NHS App | 3b | 2 |  |
| NHS Online Bexley | 2 | 2 | 2 |
| Nujjer | 3a | 2 | 3a |
| OurPath | 2 | 3a | 3a |
| Oviva | 2 | 3a | 3a |
| Owise breast cancer | 2 | 2 | 3a |
| Patients Know Best | 2 | 2 | 2 |
| Peanut | 2 | 2 | 2 |
| Pzizz | 2 | 3a | 3a |
| Rafi-Tone | 3a | 3a | 3a |
| SilverCloud | 3b | 3b | 3b |
| Sleepio | 3a | 3a |  |
| Sleepstation | 3a | 3b | 3b |
| SOS QR | 2 | 2 | 2 |
| SpineWise | 3a | 2 |  |
| Squeezy | 2 | 3a | 2 |
| Squeezy CF | 3a | 3a | 2 |
| Squeezy for men | 2 | 3a | 2 |
| Stress & Anxiety Companion | 3a | 3b | 3a |
| Student Health App | 2 | 2 | 2 |
| Sugarmedown | 2 | 3a |  |
| Talk Around It Home | 3b | 3b | 3b |
| Thrive | 3a | 3a | 3a |
| Untire: Beating cancer fatigue | 3b | 3a |  |
| vCreate | 1 | 2 | 2 |
| WaitLess | 2 | 1 | 2 |
