# Supplementary material for: Medical Mobile App Classification Using the National Institute for Health and Care Excellence Evidence Standards Framework for Digital Health Technologies: Interrater Reliability Study
Source: J Med Internet Res. 2020 Jun 5;22(6):e17457. doi: 10.2196/17457 (PMC7305556; doi:10.2196/17457)
Supplement: Multimedia Appendix 2 [file jmir_v22i6e17457_app2.docx]

## Multimedia Appendix 2

Inter-rater reliability crosstables for coders 1 and 2 vs Independent

|  | |  | | Independent | |  | |  | |  | |
| --- | --- | --- | --- | --- | --- | --- | --- | --- | --- | --- | --- |
|  | |  | | **1** | | **2** | | **3a** | | **3b** | |
| Coder 1 | | **1** | | 0 | | 1 | | 0 | | 0 | |
|  | | **2** | | 0 | | 23 | | 6 | | 0 | |
|  | | **3a** | | 0 | | 3 | | 18 | | 2 | |
|  | | **3b** | | 0 | | 1 | | 0 | | 4 | |
|  | |  | |  | |  | |  | |  | |
|  | |  | | Independent | |  | |  | |  | |
|  | |  | | **1** | | **2** | | **3a** | | **3b** | |
| Coder 2 | | **1** | | 0 | | 3 | | 0 | | 0 | |
|  | | **2** | | 0 | | 20 | | 4 | | 1 | |
|  | | **3a** | | 0 | | 3 | | 12 | | 0 | |
|  | | **3b** | | 0 | | 0 | | 8 | | 5 | |
